# Surgical Margin of Excision in Basal Cell Carcinoma: A Systematic Review of Literature

**DOI:** 10.7759/cureus.9211

**Published:** 2020-07-15

**Authors:** Sohail J Quazi, Nida Aslam, Hajra Saleem, Jawaria Rahman, Safeera Khan

**Affiliations:** 1 Plastic Surgery, California Institute of Behavioral Neurosciences & Psychology, Fairfield, USA; 2 Plastic and Reconstructive Surgery, Hamad Medical Corporation, Doha, QAT; 3 Dermatology, California Institute of Behavioral Neurosciences & Psychology, Fairfield, USA; 4 Family Medicine, California Institute of Behavioral Neurosciences & Psychology, Fairfield, USA; 5 Pathology, City of Hope Comprehensive Cancer Center, Monrovia, USA; 6 Internal Medicine, California Institute of Behavioral Neurosciences & Psychology, Fairfield, USA

**Keywords:** non-melanoma skin cancer, basal cell carcinoma, narrow excision margin

## Abstract

Skin cancer is one of the most common cancers in the world and consists of melanoma and non-melanoma skin cancer (NMSC). Basal cell carcinoma (BCC) and squamous cell carcinoma (SCC) are the most common non-melanoma skin cancers. The ideal surgical treatment for BCC is complete removal, and it can be achieved either with safety margins or with micrographic control. The currently accepted treatment for basal cell carcinoma is an elliptical excision with a 4-mm surgical margin of clinically normal skin. However, because of cosmetic and functional constraints on the face, a 4-mm surgical margin is often not feasible.

We used PubMed, PubMed Central (PMC), and Google scholar as our main databases to search for the relevant published studies and used "Basal cell carcinoma" and "narrow excision margins" as Medical Subject Headings (MeSH) keywords. Fifteen studies were finalized for the review, which included 3843 lesions. The size of the lesions ranged from 3 to 30 mm, with a mean size of 11.7 mm. Surgical margins varied from 1 to 5 mm. This review was done to evaluate if small, well-defined primary BCCs can be excised using narrow surgical margins.

Based on the reviewed literature, we found that for primary well-demarcated BCCs smaller than 2 cm, in the low-risk group, a safety margin of 3 mm gives satisfactory results. In the high-risk group, and for lesions larger than 2 cm, a 4-6 mm margin is suggested for getting clear margins. Mohs micrographic surgery is advocated for more complex and recurrent lesions where the clinical margin is not apparent. However, micrographic surgery is not readily available in many places and requires more training and experience.

Therefore, excision with 2 mm margins for clinically well-defined lesions with close follow-up can be followed to preserve the healthy tissue in anatomic constraint lesions and avoid the need for complex reconstructive procedures.

## Introduction and background

Skin cancer is one of the most common cancers in the world and consists of melanoma and non-melanoma skin cancer (NMSC). Non-melanoma skin cancers are further divided into basal cell carcinoma and squamous cell carcinoma. Basal cell carcinoma (BCC) accounts for nearly 80% of the non-melanoma skin cancers [1]. 

Ultraviolet (UV) radiation is considered as a major risk factor for the development of BCC, particularly the UV B spectrum (290 to 320 nm), which causes mutations in tumor suppressor genes. The other risk factors include old age, male sex, smoking, fair skin types I and II, arsenic exposure, and immunosuppression [2].

The incidence of basal cell carcinoma increases with age and is far more common in persons aged 55-70 years, this is possibly due to the accumulative effect of extended sun exposure, along with a reduced capacity to heal DNA damage and mobilize an immunological response. A lag period of 20 to 50 years is found between the time of UV exposure to the appearance of a tumor. There has been an increase in the incidence of BCC in young adults, too, possibly as a result of increased sun exposure. In children and young adults, BCC is associated with genetic syndromes such as Bazex syndrome, basal cell nevus syndrome (also called Gorlin syndrome), and xeroderma pigmentosum [3].

The sun‐exposed areas of the head and neck are the commonly affected sites for BCC. The tumor grows slowly and behaves in a non-aggressive fashion. The tumor inﬁltrates tissues through the irregular growth of subclinical ﬁnger-like outgrowths in a three-dimensional fashion, which remains continuous with the main tumor [4].

Metastasis in BCC is rare, and it mainly causes local tissue invasion and destruction, especially on the face, head, and neck. Although, some cases may develop aggressive features such as perineural invasion (fewer than 0.2% of cases) and metastatic disease. Perineural invasion occurs when malignant cells invade in the perineural space of nerves and it must be treated with aggressive surgical excision and radiotherapy [5].

There are three goals of treatment for BCC: (1) to excise the tumor completely so that there is no recurrence of tumor at a later time, (2) to avoid any functional impairment resulting from excision of the tumor, and (3) to provide the best possible cosmetic outcome, especially for the lesions that are on the face [6].

The common treatment modalities employed for the removal of BCC are curettage, electro-desiccation, topical chemotherapy (5-fluorouracil and imiquimod), radiotherapy, standard surgical excision, and Mohs micrographic surgery (MMS) [7]. Surgical excision with complete removal of the lesion, achieved either with safety margins or with micrographic control is considered to be the most effective treatment for BCC [8]. The aim is to prevent tumor progression or recurrence which is likely to cause further tissue destruction.

The currently accepted treatment of basal cell carcinoma is an elliptical excision with a 4-mm surgical margin of clinically normal skin [9]. But a 4-mm surgical margin is often not possible because of cosmetic and functional constraints on the face. Therefore, for small, well-defined, pigmented lesions, and lesions located in more cosmetic and sensitive areas, a narrow safety margin is recommended.

This article presents a systematic analysis from various studies to provide a better basis for determining the standard surgical margin. We propose that for treatment of small, well-defined BCCs arising in the head and neck, a narrow excision margin of 3 mm is adequate, and a wider margin is needed for large aggressive and recurrent lesions.

## Review

Materials and methods

**Figure 1:**
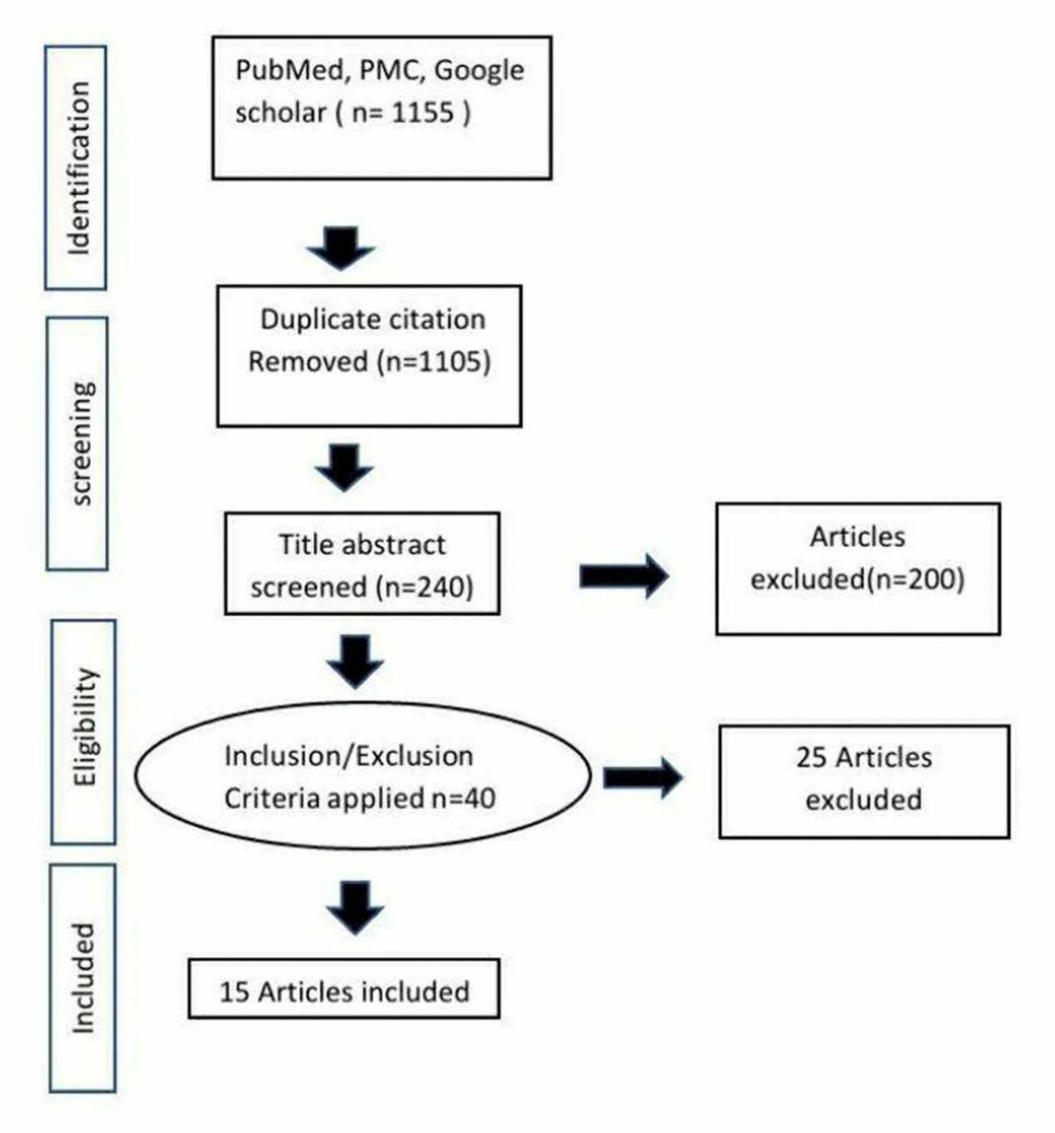
PRISMA diagram

We conducted a systematic review based on the Preferred Reporting Items for Systematic Reviews and Meta-Analyses (PRISMA) guidelines [10]. We used the databases of PubMed, PubMed Central (PMC), and Google scholar for the relevant published studies. We used "Basal cell carcinoma" and "narrow excision margins" as Medical Subject Headings (MeSH) keywords both alone and in combination to search for published papers. We screened papers from 1980 till April 2020 in the English language, and research papers in other languages, as well as duplicate papers, were removed. Other commonly used non-surgical options, like cryotherapy, topical chemotherapy, radiotherapy, and curettage for superﬁcial BCC, were not included in this paper. The selected articles were all peer-reviewed, whereas grey literature and non-peer-reviewed articles were not included in the study. After careful consideration, 40 articles were selected for the review. The papers included were assessed for quality by the New-Castle Ottawa Scale checklist to exclude as much bias as possible. After quality assessment, and further review of reported data for redundancy, a total of 15 articles were selected that had usable data for this review, and 25 articles were excluded from the paper. The inclusion criteria selected articles with original surgical excision data, histopathology data, and case series of surgical excisions. Exclusion criteria had articles with re-resection data, systematic reviews, and data involving updates in management or different techniques for excision that had no comparable analytical data. The PRISMA diagram is shown in the following figure (Figure 1).

Results

We analyzed 15 studies totaling the number of lesions to 3843. Each article was analyzed regarding histological subtype, site and size of the tumor, surgical margins, cure rates, and follow-up period. In the study, the size of the lesions ranged from 5 mm to 30 mm (average size: 11.7 mm). Surgical margins varied from 1 to 5 mm, with an average of 3.5 mm. The age of the patients ranged from 25-95 years, with the mean age of 69.7 years. In the present study, it was found that patients who underwent excision with 5 mm, 4 mm, 3 mm, and 2 mm showed a complete excision rate of 94.73%, 92.22%, 90.34%, and 88.15% respectively. The recurrence of BCC was mentioned in eight of the total studies, while the other studies did not mention the recurrence rates, and no statistically significant conclusion could be done from this review regarding the recurrence rate. Data from the studies were grouped and are presented in Table 1.

**Table 1:** Data from the studies with the title and conclusion

Author and year of publication	Type of study	No. of patients	Purpose of the study	Result/conclusion
Ito et al., 2015 [11]	Observational study	218	Narrow excision margin is a reliable method for excision of well-demarcated, primary pigmented basal cell carcinoma	Surgical removal with a 2-3-mm excision margin is an adequate treatment for well-defined, primary pigmented BCC, with a 99% complete removal rate.
Lin et al., 2016 [12]	Retrospective study	143	Treatment of pigmented basal cell carcinoma with 3 mm surgical margin in asians	The study suggests that a 3mm margin is reliable for the excision of pigmented BCC. Nonpigmented BCC had a higher risk for recurrence and thus needs careful follow up.
Ünverdi et al., 2020 [13]	Observational study	113	Recommended surgical margins for basal cell carcinoma Is 3 mm safe enough?	A 3-mm surgical margin is sufficient for BCC excision.
Thomas et al., 2003 [14]	Prospective study	150	Excision margins for non-melanotic skin cancer (NMSCS)	The majority of NMSC of up to 20 mm in diameter should be excised with a 4-mm surgical margin of skin that appears clinically healthy under magnification
Laloo et al., 2000 [15]	Prospective study	63	Head and neck basal cell carcinoma: treatment with a 2‐mm clinical excision margin	clinical excision margin of 2mm is adequate for the treatment of simple, well defined BCCs arising in the head and neck
Konopnicki et al., 2016 [16]	Retrospective study	127	Nasal basal cell carcinomas. Can we decrease surgical margins to 3 mm with a complete rate of excision?	Three-millimeters margins could be used to manage nasal BCC in selected cases.
Chadha et al., 2009 [17]	Retrospective study	90	Small margin excision of periocular basal cell carcinomas. British Journal of Ophthalmology,	Histological clearance rates and recurrence rates are not compromised by using smaller (2 mm) than conventional margins for clinically well-defined nodular BCCs
Huang et al., 2004 [18]	Prospective study	55	Small margin excision of periocular basal cell carcinoma	A small margin of excision of nodular adnexal BCCs with a delayed repair is a safe and efficient method.
Santiago et al., 2019 [19]	observational study	306	How wide should he excision margins for facial small aggressive basal cell carcinoma be? Experience with 306 Cases.	In small facial primary BCCs with aggressive histological patterns a 4 mm resection margin was enough to eradicate the lesion completely in 99% of cases
Nazhad et al., 2006 [20]	Prospective study	50	Safety margin in excision of basal cell carcinoma.	Excision margin of 4 mm is enough
Nemet et al., 2006 [21]	Retrospective study	485	Management of periocular basal and squamous cell carcinoma: A Series of 485 cases	Two-millimeter margins is adequate in preventing recurrences for nodular BCCs, and 4 mm for preventing recurrence in other types of BCC.
Wolf et al., 1987 [22]	Observational study	117	Surgical margins for basal cell carcinoma	It concluded that a minimum margin of 4 mm excision margin is necessary to eradicate a tumor in 95% of BCCs measuring less than 2 cm
Griffiths et al., 1999 [23]	Prospective study	1392	Audit of histologically incompletely excised basal cell carcinomas: recommendations for management by re-excision.	The incomplete excision in the histological report in basal cell carcinomas excised with a 3mm margin, was found in 7%of the lesions
Blomqvist et al., 1982 [24]	Observational study	477	Surgical results in 477 basal cell carcinomas.	The study concluded that 3mm margins eradicated the tumor in only 85% of cases
Kimyai-Asadi et al., 2005 [25]	Observational study	134	Efficacy of narrow-margin excision of well-demarcated primary facial basal cell carcinomas	Narrow excision margin of 1-3 mm is not enough for the removal, of well-defined, primary nodular BCCs of the face.

Discussion

The first description of a basal cell carcinoma was by Arthur Jacob, an Irish ophthalmologist in 1827, who authored the term "rodent ulcer". The rodent ulcer in his article later came to be known as basal cell carcinoma (BCC), as histologically, it looked like the basal cells of the epidermis. It is currently believed that pluripotential stem cells have a high potential to convert to basal cell carcinoma when exposed to excessive sunlight or have p53 gene mutations [26].

Pathophysiology of Basal Cell Carcinoma

BCCs are composed of several different histopathologic subtypes that can look and behave differently. These include nodular, superficial spreading, morpheaform, and pigmented. Nodular type is the most common subtype of BCC and consists of a nodule with central elevation and overlying ulceration. Superficial spreading appears as a scaly irregular plaque with micro erosions and is commonly seen in young adults. The morpheaform type is also known as sclerosing or infiltrating basal cell skin cancer. It has wide subclinical extension and may infiltrate cutaneous nerves and are therefore known to be aggressive tumors [27].

In 2006, Crowson divided BCC into indolent and aggressive types depending on the histological subtype. Indolent BCC includes superficial and nodular subtype, whereas aggressive BCC includes infiltrative, metatypical, micronodular, and sclerosing. It has been shown in various studies that aggressive tumors were associated with increased subclinical extension, indicating that aggressive tumors require wider surgical margins [28]. Based on the size and histology type, the BCC is divided into high risk and low-risk groups. BCCS larger than 2 cm in size may demonstrate wider subclinical invasion; therefore, lesions larger than or equal to 2 cm in either width or length are considered in the high-risk groups. Recurrent lesions and aggressive subtypes like morpheaform are also grouped in high-risk groups, whereas lesions smaller than 2 cm in size, nodular, and superficial spreading subtypes are included in the low-risk groups [28].

Management Options: How Much Excision Is Enough?

The National Comprehensive Cancer Network (NCCN) guidelines categorized BCC as low and high risk, based on the histological subtype, size, and risk of recurrence. For well-defined, low-risk tumors, NCCN recommends 4 mm peripheral margins to achieve a complete excision rate of 95 % whereas, for high-risk lesions, 4 to 6 mm peripheral margins are suggested [29]. The European Dermatology Forum (EDF) guidelines on surgical excision margins of BCC recommends 3 to 4 mm peripheral margins for low-risk BCC, and 5 to 10 mm peripheral margins for high-risk BCC. In contrast, according to the international guidelines, it recommends a safety margin of 3 millimeters for low-risk BCC to achieve complete excision in 85% cases, and the rate of incomplete excision is found to be 15%. [30].

The adequate treatment of BCC is considered to be complete resection with clear margins. But sometimes, a microscopic extension of the tumor can occur beyond the clinical margins. Therefore, surgical treatment aims to remove both the clinically visible tumor and its microscopic extension into the surrounding normal-appearing skin. This can be achieved by excising the tumor along with a margin of clinically normal skin. It is known that the subdermal adipose tissue is resistant to spread so, it is important to excise BCC to the level of subdermal adipose tissue [31].

Mohs micrographic surgery is the most reliable procedure to completely remove the tumor and achieve high cure rates for both primary and recurrent lesions. It has been found in many studies that a 99% cure rate can be achieved by using a surgical margin of 1 or 2 mm of normal skin through Mohs micrographic surgery [32]. However, micrographic surgery is not readily available in many places and requires more training and experience besides being more time-consuming [33]. Hence, Mohs micrographic surgery is advocated for more complex lesions where the clinical margin is not apparent, and for recurrent lesions.

The use of an elliptical 4 mm excision margin on the face is often not feasible, especially for lesions over the eyelids, nose, and ears because of anatomic and cosmetic constraints. Therefore, in low-risk groups, some authors recommend the use of narrow excision margins of 3 mm to decrease the size of the defect [34]. This also decreases the size of the postoperative scar and avoids the need for flaps or grafts to close the defect.

A review of the literature on the surgical treatment of basal cell carcinoma was performed to establish an algorithm for the management of basal cell carcinoma, which could assist in the choice of surgical procedure and decide on surgical margins, taking into account the major factors which influence complete cure. This study was done to evaluate if small, well-defined primary BCCs can be excised using narrow surgical margins.

In a recent study done in Japan in 2014 by Ito et al., out of 288 patients, 218 (75.7%) underwent excision with a narrow margin (≤3 mm), and 60 patients (24.3%) had lesion excised with a wide margin (≥4 mm). Two cases (0.7%) that were excised with a narrow (≤3 mm) margin were found to be margin positive. Complete removal rates were found in 95.7% (44 of 46 lesions) in the ≤2-mm group and 100% (172 of 172 lesions) in the 3-mm group. The remaining two patients (0.7%), who had excision with 2-mm margins, were associated with tumor-positive margins. Thus, they concluded a 95.3% cure rate for a 2 mm margin and a 100% cure rate for a 3 mm margin. However, these studies lack sufficient data on long-term outcomes [11].

 In 2016, Lin et al. reported his findings on 143 patients. They used a 5-year follow-up design to determine whether a 3 mm surgical margin was appropriate for excision of pigmented and non-pigmented BCC, and they used recurrence as the outcome measurement. They concluded that a 3 mm margin is adequate for excision of pigmented BCC, but non-pigmented BCC had a higher risk of recurrence and thus needs careful follow up [12].

A similar study by Univerdi et al. 2020 analyzed patients from 2016 to 2018. Ninety-nine lesions from 91 patients with BCC not exceeding 2 cm in size were included and excised with 3 mm (n = 53) or 5 mm (n = 46) surgical margins. Three of the fifty-three lesions that were excised with 3 mm surgical margins had a positive surgical margin, whereas the 46 lesions excised with 5 mm margins were all completely excised. Hence, they concluded that a 3 mm margin is safe for BCC excision [13].

 In support of these findings, another study by Thomas et al. operated 92 cases of BCC that were excised by 1 mm to 4 mm surgical margins. The data was assessed to form the suitability of 1-, 2-, 3-, or 4-mm surgical excision margins. It was analyzed that a 4-mm surgical margin gave clear margins in 96% of cases of basal cell carcinoma, and they suggested a 4 mm margin for aggressive BCCs and a 3 mm margin for well-demarcated BCCs [14].

Laloo and Sood published a study in the year 2000, analyzed that out of 63 patients having a well-demarcated BCC of the head and neck that were excised with 2-mm margins, 60 (95%) had histologically clear margins [15]. Konopnicki et al., in their publication, selected 132 patients with 145 lesions on the nose that were excised by the plastic surgeons over a period of three years. Of the 132 patients, 17 cases lacked data and were excluded from the study, and 115 patients with 127 lesions were included. Among the 127 lesions, 32 were resected with 3 mm surgical margins (group 1), 45 with 4 mm (group 2), and 50 with 5 mm (group 3). The incomplete excision rates were found to be 4 (12.5%), 10 (22.2%), and 8 (16%) with group 1 (3 mm), group 2 (4 mm), and group 3 (5 mm) respectively. They concluded that 3 mm surgical margins could be used to treat nasal BCCs as there was no significant difference in incomplete excision rates between the three groups, i.e., 3 mm, 4 mm, and 5 mm [16].

Chada et al. did a study on ninety cases with periocular basal cell carcinoma that underwent excision with 2 mm margins. Out of the 90 patients, 78 (86.7%) tumors were found to be excised entirely after the first surgery. Of the 12 inadequately excised tumors, eight were in the clinical margin-controlled group (i.e., the group which underwent primary direct closure). The drawback of the study was that it was retrospective, and there was no data on the size of the tumors excised [17].

Hsuan et al. selected 55 patients with primary nodular lesions who underwent excision with 2 mm margins. The reconstruction was done after the result of formal paraffin sections came negative for margin involvement (i.e., two-stage surgery). Ten of their excisions were reported to have incomplete margins and underwent a further 2 mm excision. Using this method, they achieved a 0% recurrence rate [18]. 

Santiago et al. researched 306 cases of BCC with an average size of 5.7 mm (range: 5-6 mm). Excision of the tumors using 2, 3, and 4 mm margins achieved complete excision of the lesion, including the subclinical extension area, in 73.9%, 94.4%, and 99% of cases, respectively. The study concluded that a 4-mm resection margin was enough to eradicate the lesion in 99% of cases of primary small facial BCCs with aggressive histological patterns [19]. Nazhad et al. did a similar study on 50 patients where all patients underwent surgical excision by one dermatologist with a 4 mm safety margin. Pathologic examination of the excised lesion showed complete excision rates in 48 patients (96%), and two cases (4%) had incomplete excision [20].

Nemet et al. analyzed 417 cases with confirmed BCC of the eyelid in a tertiary referral eye center in Sydney, Australia. Excision was done for all cases after marking skin under tension with margins of 3 mm. Incomplete excision was found in 106 (25.4%) cases, and 311 cases had complete excision. The rate of incomplete excision was significantly higher in the morpheaform subtype compared with the nodular subtype of BCC. Thus, they concluded that the 2 mm margin is adequate in preventing recurrences for nodular BCCs, and 4 mm for morpheaform types of BCC [21]. 

Wolf and Zitelli used Mohs micrographic surgery with horizontal frozen sections and showed that for well-demarcated primary BCCs less than 2 mm in diameter, a 4 mm safety margin is necessary to remove the tumor completely in more than 95% of cases. They defined the margins of BCC by inspection under bright theatre lights and palpation and concluded that 3 mm margins eradicated the tumor in only 85% of cases [22].

In an audit done by Griffith et al., 1392 basal cell carcinomas were excised with a 3 mm margin, over 10 years. The incomplete excision in the histological report was found in 99 (7%) of the lesions [23]. In another study done in 1982 in Sweden by Blomqvist et al., 477 BCCs were excised with macroscopic margins of at least 3 mm. When the margins of the specimens were not microscopically free from the tumor, immediate re-excision was carried out. Recurrence was defined as a tumor occurring within a 10 mm radius from the scar. Microscopically inadequate margins were found in 20 cases (4.19%), and a re-excision was performed. Only 11 (2%) of the tumors recurred. Most recurrences appeared within six months [24]. 

In contrast, to the above studies, Kimyai-Asadi et al. reported that of 134 well-defined primary BCC, excisions with 1-, 2- and 3-mm margins positive margins were found in 16%, 24% and 13% of tumors, respectively. They concluded that narrow-margin elliptical excision with margins up to 3 mm is only 80% effective in clearing these tumors. This is well below the 95% standard for tumor clearance that is expected in cutaneous surgical oncology. However, this study had a small sample size and surgery done by only one surgeon and could have led to a bias in the selection of cases [25].

A comparison of the excision margin in different studies is made in the table below (Table 2).

**Table 2:** Comparison of excision margin in different studies

Study	Total number of cases	Excision margin	Number of skin lesions	Complete excision	Incomplete excision	Recurrence
Ito et al. [11]	288	2mm	46	44	2	0
		3mm	172	172	0	
		4mm	17	17	0	
		5mm	53	53	0	
Unverdi et al. [12]	99	3mm	53	50	3	-
		5 mm	46	46	0	
Lin et al. [13]	143	3mm	143	120	23	11
Thomas et al. [14]	92	4mm	92	87	5	-
Sood et al. [15]	63	2 mm	63	60	3	0
Konopnicki et al. [16]	127	3 mm	32	28	4	-
		4mm	45	35	10	
		5mm	50	42	8	
Chada et al. [17]	90	2mm	90	78	12	3
Hsuan et al. [18]	55	2mm	55	45	10	0
Santiago et al. [19]	306	2mm	226	206	20	-
		3mm	63	54	9	
		4mm	14	11	3	
		5mm	3	3	0	
Nazhad et al. [20]	50	4mm	50	48	2	-
Nemet et al. [21]	417	3mm	417	311	106	27
Wolf et al. [22]	117	4mm	117	111	6	-
Griffith et al. [23]	1392	3mm	1392	1293	99	40
Blomqvist et al. [24]	477	3mm	477	457	20	11
Kimyai-Asadi et al. [25]	134	1mm	25	19	4	-
		2mm	85	65	20	
		3-mm	24	21	3	

In a systematic review by Luz et al., it was found that the use of 4-mm margins was satisfactory for well-defined primary BCCs smaller than 2 cm in diameter. They discovered that 3 mm margins could give similar cure rates, and sometimes a 2 mm margin may be enough to remove the BCC completely. But they reported increased recurrence risk with safety margins smaller than 3 mm, even though histopathology is tumor-free. They also recommended that for recurrent lesions, surgical techniques with micrographically controlled margins exceeding 6 mm are more appropriate [35].

In a similar meta-analysis by Yusuf et al., the total number of patients analyzed was 10,261. They compared different surgical margins with complete excision rate and concluded that for those surgeons who desire a minimum 95% cure rate, 3-mm surgical margin could be safely used for basal cell carcinoma lesions 2 cm or smaller [36].

In the present study, it was found that with increasing peripheral surgical margins, the rate of complete excision was increased and was found to be 88.15% for 2 mm safety margin, 90.34% for 3 mm margin, 92.22% for 4 mm margin, and 94.73% for 5 mm. The rate of incomplete excision was less with the 5 mm group (5.76%) compared to the 2 mm group (11.85%). The recurrence rate was mentioned in eight out of the total studies and included 2925 lesions in which 92 (3.14%) lesions had recurrences. Most of the patients with recurrence had lesser than 3 mm as the margin of excision. As more details about the recurrence were not found in the studies, any statistically significant conclusion regarding the recurrence rate was not made from this review. The following table shows the percentage of complete and incomplete excision with different surgical margins (Table 3).

**Table 3:** Percentage of complete and incomplete excision with different surgical margins

Margin	Completely excised	Incompletely excised	Total number of cases	Percentage of completely excised	Percentage of incompletely excised
1mm	19	6	25	76%	24%
2mm	498	67	565	88.15%	11.85%
3mm	2499	267	2766	90.34%	09.65%
4mm	309	26	335	92.22%	07.76%
5mm	144	8	152	94.73%	05.26%
Total	3469	374	3843		

**Figure 2:**
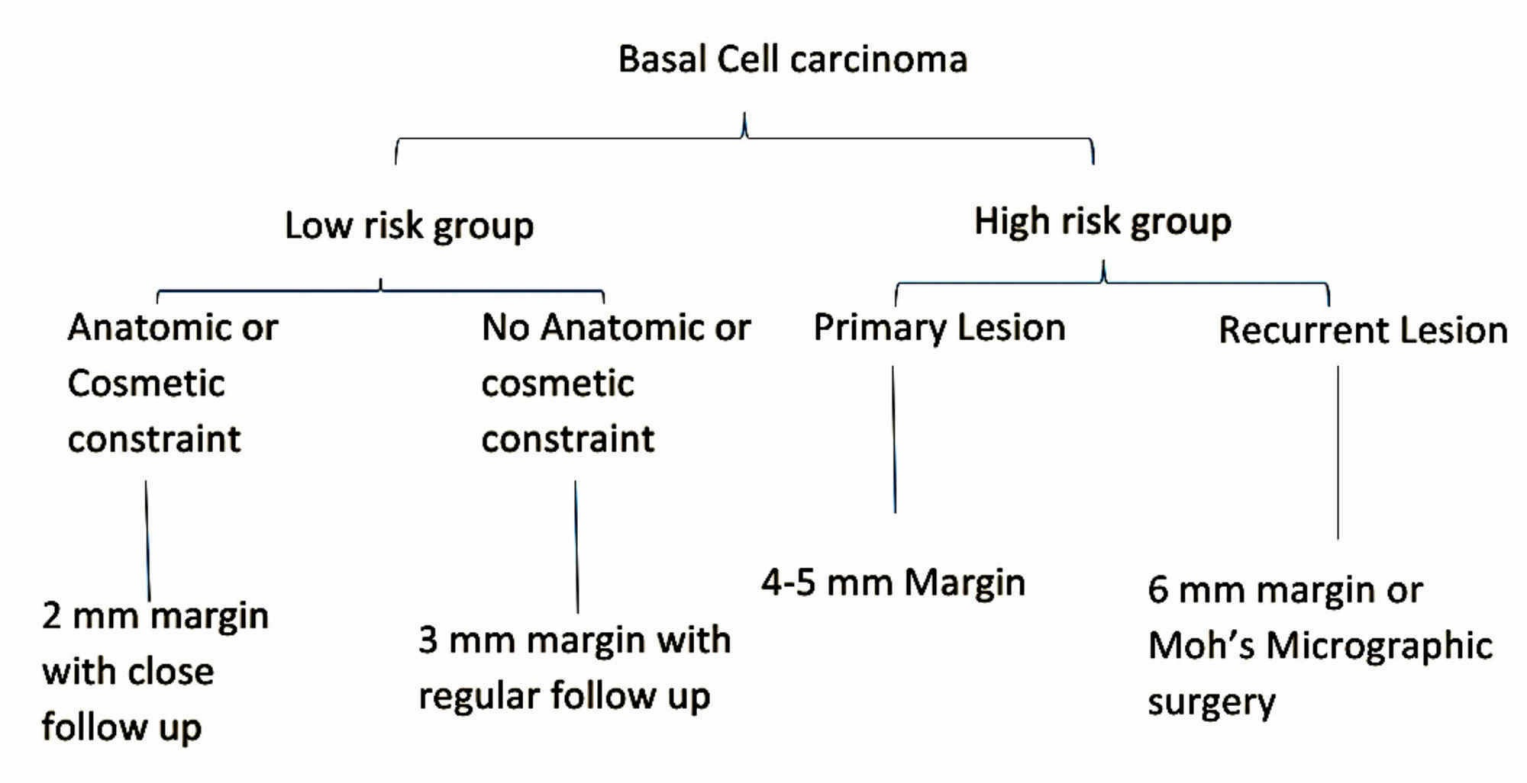
Algorithm for surgical margins in different types of BCC

Based on reviewed literature and their comparison, it can be concluded that for low-risk BCC (<2 cms or nodular or superficial spreading), a safety margin of 3 mm margins gives a satisfactory result. However, in certain situations where the wide margin is not feasible because of anatomic and cosmetic constraints, a 2 mm margin with close follow up can be followed in low-risk groups. For lesions in high-risk groups (>2 cms or morpheaform or infiltrative), a 4 mm margin is adequate for getting clear margins. But in recurrent lesions, Mohs micrographic surgery or an excision margin of 6 mm is recommended. Based on this interpretation, an algorithm is suggested for low-risk and high-risk BCC depending on the size and histological subtype of BCC. The algorithm for the surgical excision margin in BCC is shown in Figure 2.

Limitations

The limitation of this study was that there were fewer studies about safety margin in large tumors with aggressive features. Another limitation of this study is that only a few studies include long term follow up and recurrence rate of the patients. Since most studies regarding the surgical margins have included small tumors, the proper approach for high-risk groups and recurrence needs further research. Although micrographic control is the best option, some authors still recommend greater than 4 mm margins for high-risk groups and 6 mm margins for recurrent BCCs as adequate. Hence, more evaluation should be done for excision margin in large and recurrent tumors regarding the feasibility of surgical margins.

## Conclusions

From the above study, we found that the rate of incomplete excision decreased with an increase in the size of the surgical margin. Hence 5 mm margin may be considered as the safest margin for all the basal cell carcinomas. But because of the anatomical constraints, it is difficult to choose a wide safety margin, especially lesions near the eyes, nose, or ear. Therefore, for basal cell carcinoma lesions 2 cm or smaller, in the low-risk group, a 3 mm surgical margin can be safely used. If anatomic constraints do not allow wide excision, a 2 mm margin can be used for small low-risk lesions with close follow up. For lesions larger than 2 cm, or in the high-risk group, a 5 mm margin seems adequate for getting clear margins. For recurrent lesions, a 6 mm margin or Mohs micrographic surgery is recommended. Mohs surgery remains the technique that results in minimum tissue excision. However, in the absence of its availability, healthy tissue in anatomic constraint lesions can be preserved using 2 mm margins for clinically well-defined lesions simplifying subsequent reconstructive procedures.
